# Brain connectivity alterations after additional sensorimotor or motor therapy for the upper limb in the early-phase post stroke: a randomized controlled trial

**DOI:** 10.1093/braincomms/fcab074

**Published:** 2021-04-12

**Authors:** Nele De Bruyn, Leen Saenen, Liselot Thijs, Annick Van Gils, Eva Ceulemans, Bea Essers, Kaat Alaerts, Geert Verheyden

**Affiliations:** Department of Rehabilitation Sciences, KU Leuven—University of Leuven, 3001 Leuven, Belgium

**Keywords:** sensorimotor therapy, randomized controlled trial, upper limb, stroke, resting-state fMRI

## Abstract

**Somatosensory function plays an important role for upper limb motor learning. However, knowledge about underlying mechanisms of sensorimotor therapy is lacking. We aim to investigate differences in therapy-induced resting-state functional connectivity changes between additional sensorimotor compared with motor therapy in the early-phase post stroke. Thirty first-stroke patients with a sensorimotor impairment were included for an assessor-blinded multi-centre randomized controlled trial within 8 weeks post stroke [13 (43%) females; mean age: 67 ± 13 years; mean time post stroke: 43 ± 13 days]. Patients were randomly assigned to additional sensorimotor (*n* = 18) or motor (*n* = 12) therapy, receiving 16 h of additional therapy within 4 weeks. Sensorimotor evaluations and resting-state functional magnetic resonance imaging were performed at baseline (T1), post-intervention (T2) and after 4 weeks follow-up (T3). Resting-state functional magnetic resonance imaging was also performed in an age-matched healthy control group (*n* = 19) to identify patterns of aberrant connectivity in stroke patients between hemispheres, or within ipsilesional and contralesional hemispheres. Mixed model analysis investigated session and treatment effects between stroke therapy groups. Non-parametric partial correlations were used to investigate brain−behaviour associations with age and frame-wise displacement as nuisance regressors. Connections within the contralesional hemisphere that showed hypo-connectivity in subacute stroke patients (compared with healthy controls) showed a trend towards a more pronounced pre-to-post normalization (less hypo-connectivity) in the motor therapy group, compared with the sensorimotor therapy group (mean estimated difference = −0.155 ± 0.061; *P* = 0.02). Further, the motor therapy group also tended to show a further pre-to-post increase in functional connectivity strength among connections that already showed hyper-connectivity in the stroke patients at baseline versus healthy controls (mean estimated difference = −0.144 ± 0.072; *P* = 0.06). Notably, these observed increases in hyper-connectivity of the contralesional hemisphere were positively associated with improvements in functional activity (*r* = 0.48), providing indications that these patterns of hyper-connectivity are compensatory in nature. The sensorimotor and motor therapy group showed no significant differences in terms of pre-to-post changes in inter-hemispheric connectivity or ipsilesional intrahemispheric connectivity. While effects are only tentative within this preliminary sample, results suggest a possible stronger normalization of hypo-connectivity and a stronger pre-to-post increase in compensatory hyper-connectivity of the contralesional hemisphere after motor therapy compared with sensorimotor therapy. Future studies with larger patient samples are however recommended to confirm these trend-based preliminary findings.**

## Teaser

Stroke induces alterations in resting-state functional connectivity consisting of both hyper- and hypo-connectivity. Therapy-induced changes show a trend towards increases in contralesional intrahemispheric connectivity for both hyper- and hypo-connected regions suggesting the supportive role of the contralesional hemisphere in recovery during the early rehabilitation phase post stroke.

## Graphical Abstract

**Graphical Abstract:**
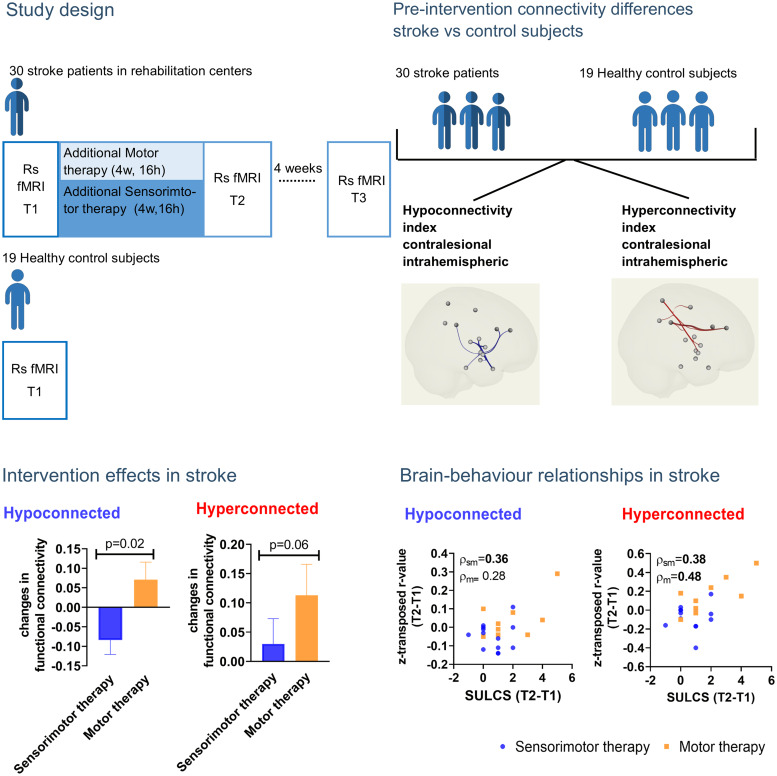

## Introduction

Stroke is generally known to affect both local and remote brain areas, thereby severely compromising brain function. In the past decade, resting-state functional magnetic resonance imaging (rs-fMRI) has emerged to investigate the effect of stroke on functional connectivity between remote brain areas.^1^ Previously, changes in resting-state fMRI (rs-fMRI) connectivity have been reported to be associated with functional outcome such as neglect or motor function. For example, lower connectivity values in patients after stroke were associated with more motor impairments or more severe neglect.^2–5^ Additionally, improvements in motor function related to an intervention or recovery have been shown to be associated with alterations in functional connectivity towards normative levels within and between functional brain networks (review, see Thiel and Vahdat^6^).

Importantly, aside motor impairments, somatosensory impairments are highly common post stroke. In the acute phase, up to 89% of the patients with upper limb impairments present with a somatosensory impairment in one or more modalities.^7^ In a study of 19 acute stroke patients, lower interhemispheric and ipsilesional intrahemispheric functional connectivity within the somatosensory network has been identified in the subgroup of patients displaying more severe compared with mild to moderate somatosensory impairments in the upper limb.^8^ Furthermore, in a longitudinal study from 1 to 6 months post stroke, improvement in touch sensation due to spontaneous recovery was shown to be associated with increased connectivity between inferior parietal cortex and middle temporal gyrus with contralesional secondary somatosensory cortex (S2), and between contralesional thalamus and cerebellum.^9^ Next to normalization of functional connectivity related to severity of impairment or to natural recovery, also changes related to therapy-induced improvements are reported in literature. A variety of different therapy approaches does exist within stroke rehabilitation, generally described under the name ‘motor’, ‘somatosensory’ or ‘sensorimotor’ therapy. The aim of these therapy approaches is to improve movement, somatosensation or the integration of both, respectively. Nevertheless, this literature mainly focusses on effect of therapy on clinical (motor) outcome and to a lesser extent on functional connectivity or somatosensory or sensorimotor outcomes.

After somatosensory therapy, improvements in somatosensory function have been reported to be associated with changes towards normalization of functional connectivity in both rodent and human stroke patients^10^ (review, see Thiel et al.^11^). However, upper limb therapy should not only be focussed on improving somatosensory or motor deficits. An integrated approach of somatosensory and motor, i.e. sensorimotor therapy may be warranted since both elements are key components of motor learning.^12–14^ In a case study with two chronic stroke patients, improved upper limb function after sensorimotor therapy was shown to be associated with a neural reorganization. This neural reorganization indicated a reduction in deactivation of contralesional pre- and post-central gyri for one patient and increased activity in contralesional postcentral gyrus, supplementary motor area and ipsilesional inferior frontal area for the other patient.^15^ Vahdat et al.,^16^ showed that in 10 patients in a late phase post stroke, a single session of robotic reaching and proprioceptive training induced significant changes in functional connectivity. In particular, increases between bilateral supramarginal gyri and ipsilesional primary motor cortex as well as between contralesional primary somatosensory cortex and bilateral supplementary motor areas, primary motor cortex and premotor cortex were shown. These neural changes were associated with improved clinical motor and somatosensory function. To date, studies have provided limited insights into sensorimotor therapy and related neural reorganizations in patients in the chronic phase post stroke. Little is known with regard to the effects of sensorimotor therapy on functional reorganizations in the earlier phase post stroke, and when directly compared with motor therapy.

Therefore, we aimed to conduct a randomized controlled trial (RCT) comparing the effect of an additional 4-week sensorimotor therapy versus motor therapy program on clinical outcomes and neural changes in functional connectivity at the level of the sensorimotor network in patients in the early rehabilitation phase, i.e. within 8 weeks post stroke. Primary outcome of our RCT was the clinical motor outcome, which is discussed in another paper.^17^ This paper will focus on the secondary resting-state functional connectivity outcome measure. We aim to investigate aberrations in resting-state functional connectivity of the sensorimotor network in stroke patients compared with healthy age-matched controls. Additionally, we aim to investigate the therapy-induced effects on these resting-state functional connectivity alterations associated with clinical improvements of function. We expected aberrant functional connectivity patterns in patients after stroke compared with healthy controls in the sensorimotor network both in and between hemispheres. Further, we hypothesize that in stroke patients, patterns towards normalization of functional connectivity will be present to a greater extent after receiving sensorimotor therapy compared with receiving motor therapy alone. Furthermore, pre-to-post normalizations in functional connectivity patterns are hypothesized to be associated with clinical improvements.

## Materials and methods

Methods of this assessor-blinded multicentre RCT are described elsewhere^18^ but a summary is provided below. Ethical approval was obtained from the ethical committee of UZ/KU Leuven (s60278) and the trial is registered at clinicaltrials.gov (NCT03236376).

### Participants

Stroke participants were recruited at admission to four rehabilitation wards in Flanders, Belgium: UZ Leuven (Pellenberg), Jessa hospitals (Herk-de-Stad), Heilig Hart Hospital (Leuven) and RevArte (Antwerp) with following inclusion criteria: first stroke and recruited within 8 weeks post stroke, sensorimotor upper limb deficit defined as <52 out of 57 on Action Research Arm Test (ARAT)^19^ and sensory composite score <0.00,^20^ aged 18 years or older and with sufficient cooperation. Patients were excluded if other neurological or musculoskeletal disorders were present affecting the upper limb, if patients had severe cognitive or communication deficits, showed contra-indications to perform magnetic resonance imaging (MRI) or did not provide informed consent. Healthy, age- and handedness-matched controls were recruited from the community. Exclusion criteria were neurological disorders, presence of somatosensory or motor impairment in the upper limb and contra-indications for MRI-scan. When an unknown neurological disorder seemed to be apparent at the research MRI, this subject was excluded from analysis anyway.

### Procedure

All stroke participants were assessed at three time points: Baseline (T1), post-intervention (T2) (4 weeks after baseline) and 4 weeks after post-intervention (T3) with an extensive test battery of motor and somatosensory outcome measures (see Table 1 for overview) and a neuro-imaging scanning protocol consisting of an anatomical and resting-state MRI scan. The MRI scan protocol was performed at the same MRI scanner at the KU Leuven, Belgium, for all subjects (healthy controls and stroke participants). ARAT at T2 was set as the primary clinical end-point. Healthy controls were assessed once using the same MRI protocol.

**Table 1:** Outcome measures

Motor	Primary:	ARAT	Action research arm test^19^: grasp, grip, pinch and gross movement of the affected upper limb
	Secondary:	FMA-UE	Fugl-Meyer motor assessment—upper extremity^21^: overall motor impairment of the affected upper limb: shoulder, arm, wrist, hand and fingers
		SULCS	Stroke upper limb capacity scale^22^: upper limb capacity
Somatosensory		Em-NSA	Erasmus modified Nottingham sensory assessment^23^: light touch, pressure, sharp, sharp-dull discrimination, position sense of the upper limb
		PTT	Perceptual threshold of touch^24^: light touch
		TDT	Texture discrimination test^25^: texture discrimination
		WPST	Wrist position sense test^26^: position sense
		fTORT	Functional tactile object recognition test^27^: recognition of object by touch

Table adapted from De Bruyn et al.^18^

### Intervention

Stroke participants were block randomized and stratified for severity of motor impairment (ability to perform wrist and finger extension against gravity or not), type of stroke (ischaemic/haemorrhagic) and presence of neglect. Patients were allocated to an experimental group receiving sensorimotor therapy or control group receiving motor therapy, both in addition to usual inpatient rehabilitation care. Both groups received 16 training sessions of 1 h over 4 weeks. Sensorimotor therapy consisted of 30 min of sensory retraining based on the SENSe training program^20^ and 30 min somatosensory integrated motor exercises such as sliding over different textures. The control group received 30 min of cognitive tabletop games and 30 min of similar motor exercises without any emphasis on somatosensation such as sliding over always the same surface of a table. A more detailed description of the therapy content can be found in our protocol.^18^

### Brain imaging data acquisition and preprocessing

Functional MRI scans (fMRI) of all subjects (stroke patients and healthy controls) were acquired on the same 3.0 Tesla Philips MR scanner (Best, the Netherlands) with a 32-channel phased-array head coil at UZ Leuven hospitals. Scan sessions consisted of anatomical images: a T_1_- and a T_2_-weighted fluid-attenuated inversion recovery imaging (FLAIR); and a 7-min rs-fMRI scan. During the resting-state scan, patients were instructed to lay relaxed in supine position (but not sleep), fixate a white cross and think of nothing in particular.

#### MRI scanning parameters

T1 anatomical scan was acquired with the following parameters: 182 coronal slices covering the whole brain, repetition time (TR) = 9.6 ms, echo time (TE) = 4.6 ms, field of view (FOV) = 250×250 mm^2^, slice thickness = 1.2 mm and no interslice gap. T2 FLAIR images were acquired with following parameters: 321 transverse slices covering the whole brain, TR = 4800 ms, TE = 351 ms, inversion time = 1650 ms, FOV = 250 × 250 mm^2^, slice thickness = 1.12 mm and interslice gap = 0.56 mm. rs-fMRI parameter settings were: TR = 1700 ms, TE = 33 ms, flip angle = 90°, FOV = 230 × 230, slice thickness = 4 mm, matrix = 64 × 62, 250 functional volumes without interslice gap, duration = 7.15 min.

#### MRI data preprocessing

For each assessment point, lesion mapping was based on both T1 and FLAIR images and performed semi-automatically using the clusterize toolbox^28^ for statistical parametric mapping (SPM) 12 combined with manual inspection and corrections in MRIcron. Preprocessing was performed using SPM12 (Welcome Department of imaging Neuroscience, London, UK) and the CONN functional connectivity toolbox 17.f^29^ implemented in MATLAB R2019b (mathworks). Preprocessing consisted of coregistration of the anatomical T1 and resting-state image to the Montreal Neurological Institute (MNI) template, coregistration of resting-state image to T1 image, realignment of the resting-state image, segmentation and normalisation. To take into account the effect of the lesion, clinical toolbox^30^ for SPM was used for segmentation and enantiomorphic normalization to the elderly brain MNI template provided into the clinical toolbox. In order to normalize the resting-state images, transformation matrix of the anatomical image was then applied to the resting-state image.

Further analyses were performed with the CONN functional connectivity toolbox 17.f implemented in MATLAB R2019b. Realignment parameters were modelled as regressors of no interest. The implemented CompCor strategy^31^ in CONN toolbox was applied to remove white matter and cerebrospinal fluid as confounds. Bandpass filtering (0.009<*f* < 0.08 Hz) was then applied on the residual time series.

Given the potential confounding effects of micro-movements on resting-state functional connectivity,^32^^,^^33^ all reported analyses were performed on ‘scrubbed’ data,^33^ censoring frames displaying more than 0.5 mm frame-wise displacement (FD) or frame-wise changes exceeding more than 50Δ% BOLD. Approximately, 35% of the frames were removed per stroke participant. At baseline (T1), significant differences in head motion were evident between healthy controls and stroke patients with higher mean FD and percentage of scrubbed frames for the stroke patients (mean difference = 0.176; SE = 0.059; *T*(43) = 2.8; *P* = 0.01 and mean difference 18.52; SE = 8.157; *T*(45) = 2.27; *P* = 0.03, respectively). However, no group differences between both therapy groups were evident at the post-intervention (T2) or follow-up session (T3) for mean FD [T2: Mean difference = 0.28; SE = 0.15; *T*(19) = 1.87, *P* = 0.08 and T3: Mean difference = 0.18; SE: 0.14; *T*(21) = 1.29; *P*  = 0.21] nor for the percentage of scrubbed volumes [T2: Mean difference = 12.78; SE = 14.05; *T*(22) = 0.91, *P* = 0.37 and T3: Mean difference = 16.87; SE = 12.30; *T*(22) = 1.37; *P* = 0.18] (see Supplementary Fig. 1). To account for potential effects of head motion, all further analyses were performed with mean FD included as a nuisance regressor. This practice has previously been shown to provide substantial additional clearing of motion-related effects.^34^

### Definition of connections-of-interest based on baseline differences between stroke and healthy control participants

Whole-brain explorative functional connectivity analyses were performed to assess between-group differences (stroke versus healthy) in baseline (T1) functional connectivity between all regions of the cortical (*n* = 92) and subcortical (*n* = 14) Harvard−Oxford atlas. Notably, regions of the cerebellum were not included since this area was not captured fully into the field of view for the majority of patients. For each region, the mean time series were extracted and bivariate correlation coefficients between the mean time series were extracted as Fisher *z*-transformed *r* values resulting in a 106×106 connection matrix. Next, these connection matrices were compared between healthy controls and stroke patients (baseline scan). Exploratory two-sided *P*-values were set at 0.01 with seed-level false discovery rate (FDR) corrections for multiple comparisons (and mean FD included as nuisance regressor).

Connections between regions of the Harvard−Oxford atlas showing significant differences (two-sided) in connectivity between healthy controls and stroke patients at baseline were selected as connections-of-interest for further exploring the differential effect of sensorimotor versus motor therapy on functional connectivity patterns. Two groups of connections could be identified: (i) connections that showed significant *lower* functional connectivity in the stroke patients compared with controls (FDR seed-level correction *P* = 0.01, two-sided), which are further referred at as *hypo-*connected (S < H); (2) connections that showed significant *higher* functional connectivity in the stroke patients compared with controls (FDR seed-level correction *P* = 0.01, two-sided), which are further referred at as *hyper-*connected (S > H). A full list of the identified regions showing connections-of-interest is provided in Supplementary Fig. 2 and Table 1a. In short, a wide-spread network of cortical and subcortical connections, both between and within the contra- and ipsilesional hemispheres were identified (see Results section). Healthy controls were only included in this analysis to define connections of interest, further analysis were performed on stroke patients.

### Accounting for stroke lesions

Stroke lesions were mapped onto the FLAIR image using the semi-automated clusterize toolbox^28^ for SPM and manually modified using MRIcron by an experienced researcher (N.D.). Lesions were accounted for during preprocessing as well as during further ROI-to-ROI analysis. During preprocessing, segmentation and enantiomorphic normalization was performed using the clinical toolbox^30^ in SPM taking into account the lesion maps during these steps. To take into account the lesions into the ROI-to-ROI analysis, voxels located into the lesion were masked out of the ROIs of the Harvard−Oxford atlas used for these analyses for each lesion individually. So the ROI-to-ROI atlas file used was subject-specific for each stroke participant.

### Calculation of functional connectivity indexes

In order to decrease multiple comparison, we have created six index values. Further detail about the rationale is provide in the discussion. For each of the selected connections-of-interest [showing aberrant (hypo- or hyper-) connectivity in the stroke versus the control sample], the mean time series were extracted for each region and bivariate correlation coefficients between these mean time series were extracted as Fisher *z*-transformed *r* values for each stroke participant and each test session. Next, it was assessed whether the applied therapies (sensorimotor versus motor) induced different pre-to-post or pre-to-follow-up changes in functional connectivity between or within the ipsi- and contralesional hemispheres. To do so (i) an interhemispheric, (ii) an ipsilesional intrahemispheric and (iii) a contralesional intrahemispheric connectivity index was calculated separately for each stroke patient at each assessment session (T1, T2, T3). The interhemispheric connectivity index was calculated as the average of the *z*-transformed *r* values of all connections between the left and right hemispheres that showed aberrant connectivity in the stroke patients, compared with healthy controls. Note that separate indices were calculated for connections displaying hypo-connectivity (S < H: number of connections included into this index: *n* = 12 connections) or hyper-connectivity (S > H *n* = 2). Intrahemispheric functional connectivity indexes were calculated as the average of *z*-transformed *r* values of all connections within the lesioned (ipsilesional) (S < H *n* = 8 connections; S > H *n* = 3) or unaffected (contralesional) (S < H *n* = 4 connections; S > H *n* = 12) hemisphere that showed aberrant connectivity in the stroke patients compared with healthy controls. Regions could be involved in both hyper- and hypoconnected connections. In this case, only connections showing hyper-connectivity with these regions were included into the hyper-connectivity index and similar for the connections showing hypo-connectivity. Note that definition of hyper- and hypo-connectivity is only based on the previous described stroke versus healthy controls analysis. (Therapy-induced) changes in connectivity indexes are used for interpretation of the treatment effect and are only indicating the direction. In order to take into account the lesion location, voxels located in the individual lesioned area (Fig. 2) were masked out of the respective regions contributing to connections-of-interest (as listed in Supplementary Fig. 1), creating an individualized region map for each respective stroke patient, depending on lesion location.

### Statistical analysis

#### Baseline characteristics

Participants and baseline characteristics of the behavioural assessments are reported as group averages, numbers and percentages. Mann−Whitney U, Chi-square and Fisher exact tests are used to assess between-group differences at baseline (healthy controls compared with stroke patients as well as between both therapy groups). Healthy controls were only included in the analysis to define connections of interest, further analysis were only performed on stroke patients.

#### Treatment-related effects

In order to investigate between-group differences in therapy-induced pre-to-post or pre-to-follow-up changes in functional connectivity, mixed model analysis with treatment (sensorimotor or motor therapy) as the between-group factor were obtained. Separate analyses were performed for assessing changes in connectivity for each index value from baseline (T1) to the post-session (T2) immediately after treatment; and for assessing changes from baseline (T1) to the follow-up session, 4-weeks post-treatment (T3). Since age and head motion (mean FD) significantly differed at baseline between therapy groups, analyses were performed with age and mean FD as nuisance regressors (note that mean FD scores were averaged over the included sessions, e.g. average over T1 and T2, in the model assessing changes from session T1 to T2).

All statistics were performed with SPSS version 26 (IBM statistics). The significance level was set to *P* < 0.05. Bonferroni correction was applied for multiple testing over three assessment times, for the six different index values. The corrected *P*-value was *P* < 0.003.

#### Association with behavioural improvements

Correlation analyses were performed to explore relationships between treatment-induced changes in connectivity indices, and clinical improvements. Spearman partial correlation analyses were performed to correct for the nuisance regressors age and mean FD. Correlation analyses are reported within therapy groups.

As reported in more detail in De Bruyn et al.^17^ We previously explored treatment effects on clinical somatosensory and motor outcome measures in the same patient sample. In short, improvements in motor function were evident immediately after treatment and were still present at 4 weeks follow-up. Between group differences were found in favour of the group receiving motor therapy, showing better motor improvements compared with the sensorimotor therapy group. No significant differences in improvements were found for somatosensory function (see Supplementary Table 2).

#### Secondary analyses

To check robustness of results, all analyses were performed with and without masking out lesioned voxels and with and without correction for age (Supplementary Results).

### Adaptations to the protocol

After trial preregistration and protocol publication, some adaptations occurred. Two additional rehabilitation centres were included due to slow recruitment. However, only a limited number of participants were recruited from these centres. Further, too many patients were not able to perform the nine hole peg test due to severity of upper limb impairment. Thus, nine hole peg test was not included in further analysis. Also, no differences were found in cognition between groups and only three patients scored impaired on the star cancellation task, therefore no subgroup analysis based on cognition or neglect were performed.

### Data availability

The data that support the findings of this study are available on request from the corresponding author (N.D.). The clinical data consisting of raw brain imaging and demographic data are not publicly available due to privacy restriction. Other data consisting of analysis scripts, preprocessing scripts and behavioural and extracted brain imaging data without demographic data are made available in openscience framework (https://osf.io/zkhp8/?view_only=28675d8015274c598b98fa110a040550)

## Results

### Participant characteristics and behavioural outcome

**Figure 1:**
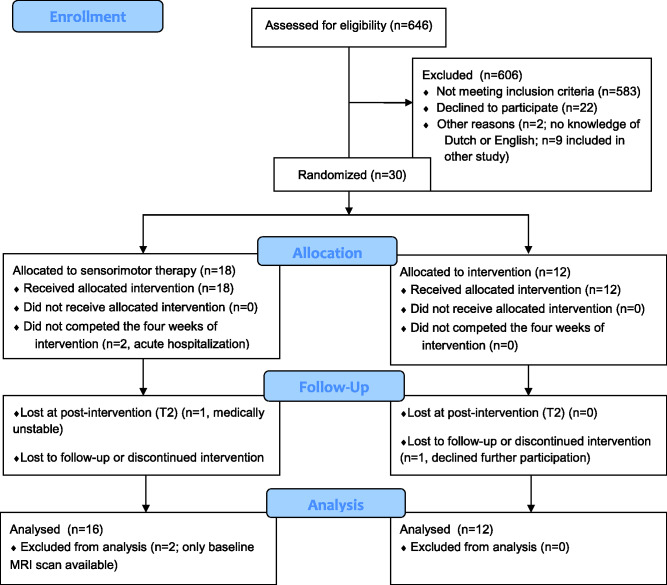
**CONSORT** 2010 flow diagram.

Between September 2017 and December 2019, 30 stroke patients were recruited to our trial and underwent the brain imaging protocol. Of these 30 patients, 18 were allocated to the sensorimotor group and 12 to the motor group. Two patients did not complete the 4 weeks of additional therapy and were lost at post-intervention (T2 and T3) due to acute sickness and readmission to the acute hospital. They were only included into the healthy versus stroke analysis. Two other patients were lost at follow-up (T3) due to acute sickness (*n* = 1) and a decline to participate (*n* = 1) (see Fig. 1 for flow diagram). The majority of the other patients that were screened were not eligible due to not having a first-ever stroke or showing other neurological or musculoskeletal disorders affecting the upper limb.

Nineteen healthy controls with mean age 65 (SD = 10) years were included of which 12 females (63%) and who were mainly right-handed (84%). No healthy controls were excluded based on unknown neurological disorder that seemed to be apparent at the research MRI scan. One healthy control was excluded due to low data quality and technical problems with preprocessing.

**Figure 2:**
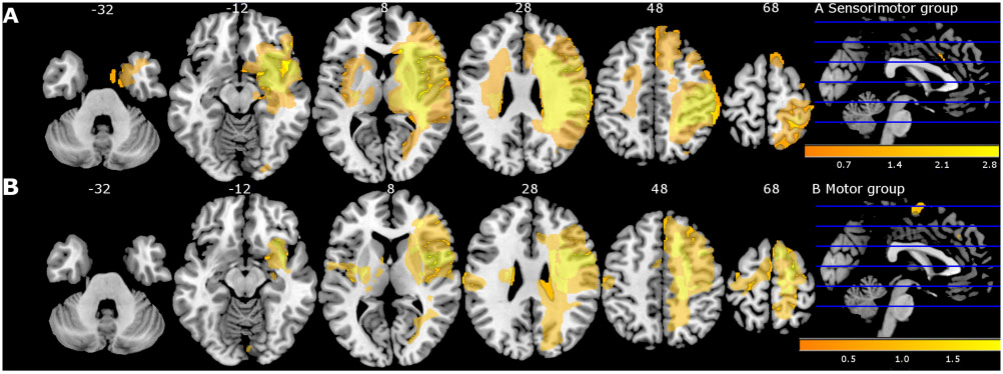
**Lesion overlay map of stroke lesion location of patients with available magnetic resonance imaging (MRI) scan (*n* = 30) displayed for sensorimotor group** (**A**) and motor group (**B**) separately. Colour indicates increasing number of patients with inclusion of that voxel into the lesion from orange to yellow (low number: orange; high number: yellow). Figure adapted from De Bruyn et al.^17^

Participant characteristics are presented in Table 2. As shown, baseline patient characteristics were overall similar across the two treatment groups except for educational level, lateralization and age. Patients of the sensorimotor group were significantly older, had lower educational level and showed more right hemisphere lesions. Baseline performance was not significantly different between both groups except for perceptual threshold of touch (PTT). The sensorimotor group showed significantly higher and thus more impaired PTT compared with motor group (*P* = 0.04). Most patients had ischaemic stroke (*n* = 23; 77%) with combined subcortical−cortical involvement. Overview of lesion location is mapped in Fig 2. Baseline differences between stroke participants and healthy controls are also presented in Table 2. As expected, significant differences were found for all sensorimotor outcome measures.

**Table 2:** Participant’s characteristics

	Sensorimotor group		Motor group		*P*-value^a^	Healthy controls		*P*-value^b^
	*n*	%	*n*	%		*n*	%	
Centre (*n*, %)								
Jessa Hospitals, Herk-de-Stad	10	33	6	20	0.56^c^			
UZ Leuven, Pellenberg	7	23	6	20				
Heilig Hart Hospital, Leuven	1	3	0	0				
Severity of motor upper limb impairment (*n*, %)								
Mild to moderate	9	30	5	17	0.71^c^			
Severe	9	30	7	23				
Age (at stroke onset) (mean, SD)	72.47 (12.45)		60.58 (10.34)		0.01^d^	65.21 (10.10)		0.47^d^
Days post stroke (mean, SD)	43.22 (12.21)		42.83 (14.58)		0.94^d^			
Gender (*n*, %)								
Male	11	37	6	20	0.72^c^	7	36.8	0.12^c^
Female	7	23	6	20		12	63.2	
Education (*n*, %)								
Lower secondary education	9	30	1	3	0.03^c^			
Higher secondary education	4	13	7	23				
Higher tertiary education - bachelor	1	3	3	10				
Higher tertiary education - master	3	10	1	3				
Unknown	1	3	0					
Type of stroke (*n*, %)								
Ischaemic	15	50	9	30	0.67^e^			
Bleeding	3	10	3	10				
Lateralization (*n*, %)								
Left hemisphere lesion	3	10	7	23				
Right hemisphere lesion	15	50	5	17	0.05^e^			
Handedness (*n*, %)								
Left	3	10	3	10	0.67^e^	3	15.8	1^e^
Right	15	50	9	30		16	84.2	
Hours additional therapy received (median, IQR)	15 (13 to 16)		16 (15–16)		0.27^c^			
**Baseline performance**								
Motor function (median; IQR)								
ARAT/57	8 (0 to 41)		6.5 (0–31)		0.72^d^			
FMA -UE/66	29 (8 to 45.5)		16 (11–39)		0.68^d^			
SULCS/10	3 (1 to 7)		3 (1–6)		0.76^d^			
Somatosensory function (median; IQR)								
Em-NSA/40	36.5 (28.5 to 39)		38 (37–40)		0.29^d^	39 (39–40)		0.04^d^
PTT	7.4 (4.7 to 9.5)		4.85 (3.6–5.8)		0.04^d^	2.5 (2.2–3.3)		0^d^
TDT-AUC	13.6 (−5.3 to 35.5)		24.0 (8.3–36.1)		0.49^d^	59.1 (48.8–69.5)		0^d^
WPST-total error (degrees)	218 (203 to 274)		317 (227–410)		0.09^d^	157 (123–258)		0.01^d^
WPST-mean error (degrees)	10.9 (10.1 to 13.7)		15.9 (11.4–20.5)		0.09^d^	8.1 (5.7–12.5)		0.01^d^
fTORT/42	31 (11.8 to 36)		37 (16.75–41)		0.08^d^	41 (39–42)		0^d^
Cognitive function (median; IQR)								
MOCA/30	22 (19.5 to 27)		25 (20.5–27)		0.47^d^			

a *P*-value indicating differences between sensorimotor and motor group.

b *P*-value indicating differences between whole stroke group and healthy controls.

c Chi-square test

d Mann−Whitney U test.

e Fisher’s exact test.

ARAT = action research arm test; AUC = area under curve; Em-NSA = Erasmus modification of Nottingham sensory assessment; FMA-UE = Fugl-Meyer assessment upper extremity section; fTORT = functional tactile object recognition test; IQR = interquartile range; MOCA = Montreal cognitive assessment; *n* = number; % = per cent given for the whole stroke group or the whole healthy group; PTT = perceptual threshold of touch; SD = standard deviation; SULCS = stroke upper limb capacity scale; TDT = texture discrimination test; WPST = wrist position sense test.

Detailed description of behavioural results from mixed models corrected for age can be found in our previous paper.^17^ In short, significant differences were found in favour of the motor group on FMA for improvements from baseline to post-intervention (*P* = 0.01) and to follow-up (*P* = 0.003). Trends towards differences in favour of the motor group were found on ARAT for improvement between all assessment time points (*P* = 0.04–0.08) and on SULCS for improvements from baseline to post-intervention (*P* = 0.08) and to follow-up (*P* = 0.02). No differences were found for somatosensory assessments (see Supplementary Table 2).

### Baseline differences in functional connectivity between stroke patients and healthy controls

**Figure 3:**
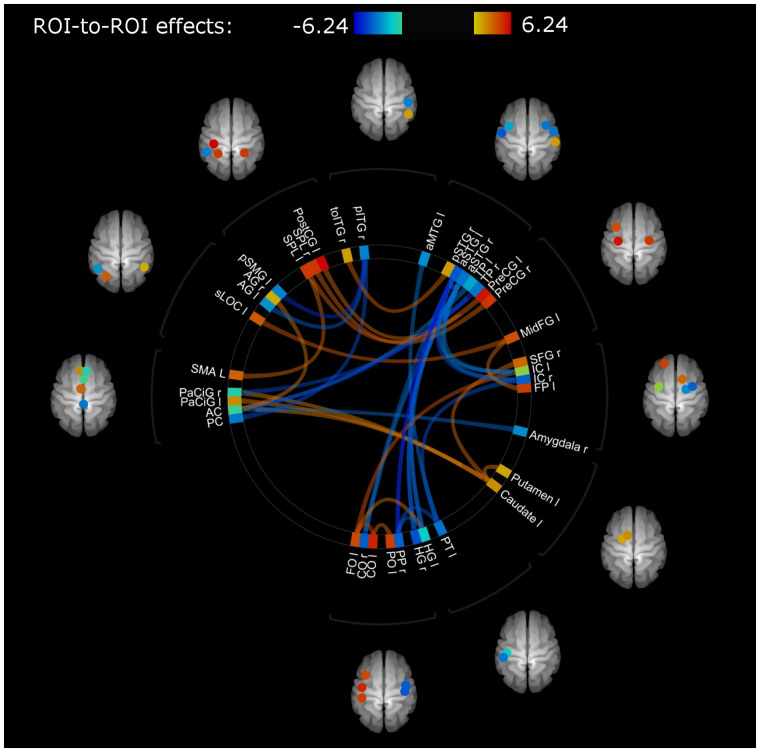
**Connections showing differences in functional connectivity between healthy and stroke participants at baseline (T1) with mean FD as regressor of no interest.** Colour of lines and ROIs indicate strength of difference in functional connectivity: Blue (lower connectivity, lowest *z*-transformed *r* value: -6.24) to red (higher connectivity, highest *z*-transformed *r* value 6.24) in stroke participants compared with healthy controls. Abbreviations of region of interest (ROI) labels can be found in Table 1. A detailed overview of involved connections and their strengths can be found in Supplemenatry Tables 3a (for hypoconnected regions) and 3b (for hyperconnected regions).

Connections for stroke patients and healthy controls at baseline that showed significantly altered functional connectivity (FDR seed-level correction *P* = 0.01, two-sided) are shown in Fig. 3 and presented in Supplementary Table 3. These ROI-to-ROI connections, showing differences at baseline, were selected for further index analysis and will be referred to as connections-of-interest showing (i) hypo-connectivity if functional connectivity was significantly lower in stroke patients compared with healthy controls (hypoconnections in stroke < healthy) and (ii) connections-of-interest showing hyper-connectivity if functional connectivity was significantly higher in stroke patients compared with healthy controls (hyperconnections in stroke > healthy).

#### Differential pre-to-post changes in functional connectivity after sensorimotor therapy versus motor therapy

Connections within the contralesional hemisphere with hypo-connectivity in stroke patients (compared with healthy controls) showed a (non-significant) trend towards normalization (less hypo-connectivity) in the motor group, whereas in the sensorimotor group a further decline of connectivity (stronger hypo-connectivity) after therapy was found (mean estimated difference = −0.155 ± 0.061; *P* = 0.02). Further, the motor therapy group also tended to show a further pre-to-post increase in functional connectivity strength among connections that already showed hyper-connectivity in the stroke patients (compared with healthy controls at baseline) (mean estimated difference = −0.144 ± 0.072; *P* = 0.06).

**Figure 4:**
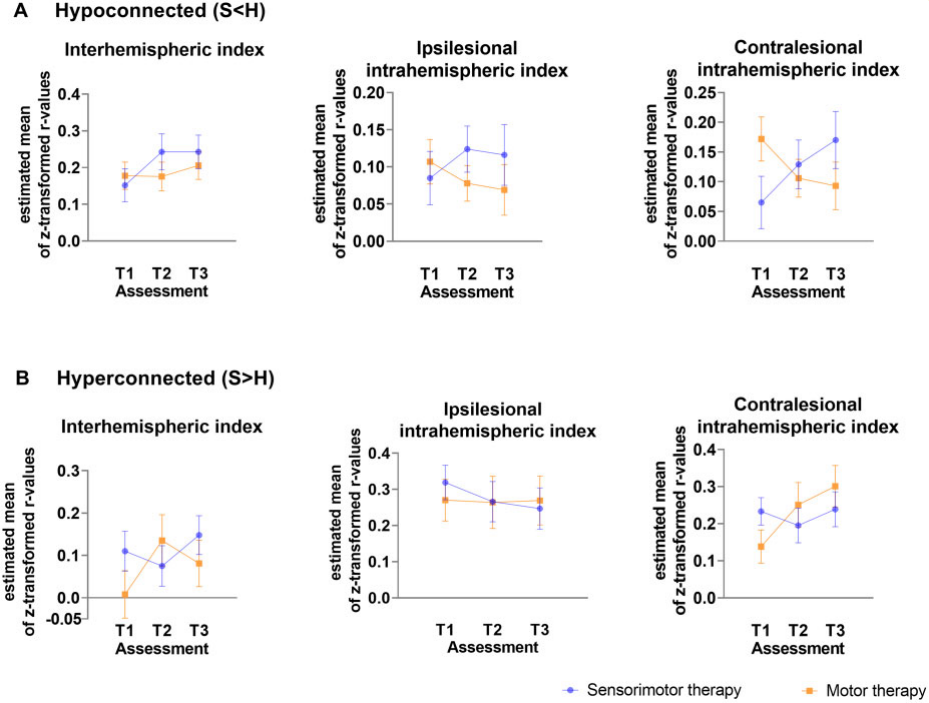
**Differences in functional connectivity between stroke patients receiving sensorimotor versus motor therapy at baseline (T1), post-intervention (T2) and follow-up (T3) for each index with age and mean FD as nuissance regressors.** (**A**) Index values of connections showing hypo-connectivity. (**B**) Index values of connections showing hyper-connectivity. Estimated marginal means with measurement error are visualized.

The sensorimotor and motor therapy group showed no significant differences in terms of pre-to-post (T1 to T2) changes in inter-hemispheric connectivity or ispilesional intrahemispheric connectivity. At the follow-up session (T3), no significant differences between therapy groups were evident. An overview of between-group differences in change scores can be found in Fig. 4 and Supplementary Table 4. All results were interpreted in the light of Bonferroni corrected *P*-value (*P* < 0.003).

### Associations between functional connectivity changes and clinical sensorimotor improvements

Brain−behaviour relationships across treatment groups were only explored for pre-to post-connectivity in the contralesional intrahemispheric connectivity indexes, since only these showed a trend towards between-group differences.

In the motor therapy group, the observed normalization of hypo-connectivity (less hypo-connectivity) in the contralesional hemisphere was non-significantly associated with clinical improvements in functional activity as measured with ARAT (hypoconnected: *r* = 0.37, *P* = 0.37, df = 6) or SULCS (hypoconnected; *r* = 0.50, *P* = 0.26; df = 5) and motor impairments as measured with FMA (hypoconnected: *r* = 0.75, *P* = 0.05, df = 5). These trend level associations might indicate reduced motor impairment in patients with stronger normalizations of hypo-connectivity. Similar, a moderate non-significant correlation was found with clinical improvements of proprioception measured with WPST (*r* = −0.67, *P* = 0.07; df = 6).

Further, pre-to-post increases in functional connectivity in the motor therapy group among connections that already showed hyper-connectivity at baseline were also associated with clinical improvements, providing indications that these patterns of hyper-connectivity were compensatory in nature. In particular, clinical improvements in functional activity (hyperconnected: *r* = 0.48, *P* = 0.005, df = 30), as assessed with SULCS were most pronounced in patients with greater pre-to-post increases in hyper-connectivity within the contralesional hemisphere. Similar moderate (non-significant) correlations were found for improvements on ARAT (hyperconnected: *r* = 0.34, *P* = 0.41, df = 6) and FMA-UE (hyperconnected *r* = 0.62, *P* = 0.14, df = 5). Only very low-to-low associations were found with somatosensory outcome measures in the motor therapy group.

While (compensatory) increases in hyper-connectivity were overall less pronounced in patients receiving sensorimotor therapy, also within this group, inter-individual differences in connectivity changes were associated with clinical improvements. In particular, patients of the sensorimotor group that showed relative pre-to-post increases in hyper-connectivity in the contralesional hemisphere also showed the strongest improvements in motor activity measured with SULCS (hyperconnected: *r* = 0.38, *P* = 0.03, df = 30) and somatosensory function as measured with Em-NSA (hyperconnected: *r* = 0.76, *P* = 0.01, df = 9) and TDT (hyperconnected: *r* = 0.54, *P* = 0.06; df = 11). In contrast, decreases in hyper-connectivity of the contralesional hemisphere were associated with improvements of stereognosis as measured with fTORT (hyperconnected: *r* = −0.69, *P* = 0.01, df = 11) within this group.

Normalization of hypo-connectivity within the sensorimotor therapy group was moderately associated with improvements in functional activity (hypoconnected: *r* = 0.45, *P* = 0.12, df = 11). Non-significant low to moderate associations were found with improvements in somatosensory function measured with Em-NSA (hypoconnected: *r* = 0.19, *P* = 0.58), PTT (*r* = −0.29; *P* = 0.34), TDT (*r* = 0.22; *P* = 0.48) and WPST (*r* = 0.57, *P* = 0.06).

**Figure 5:**
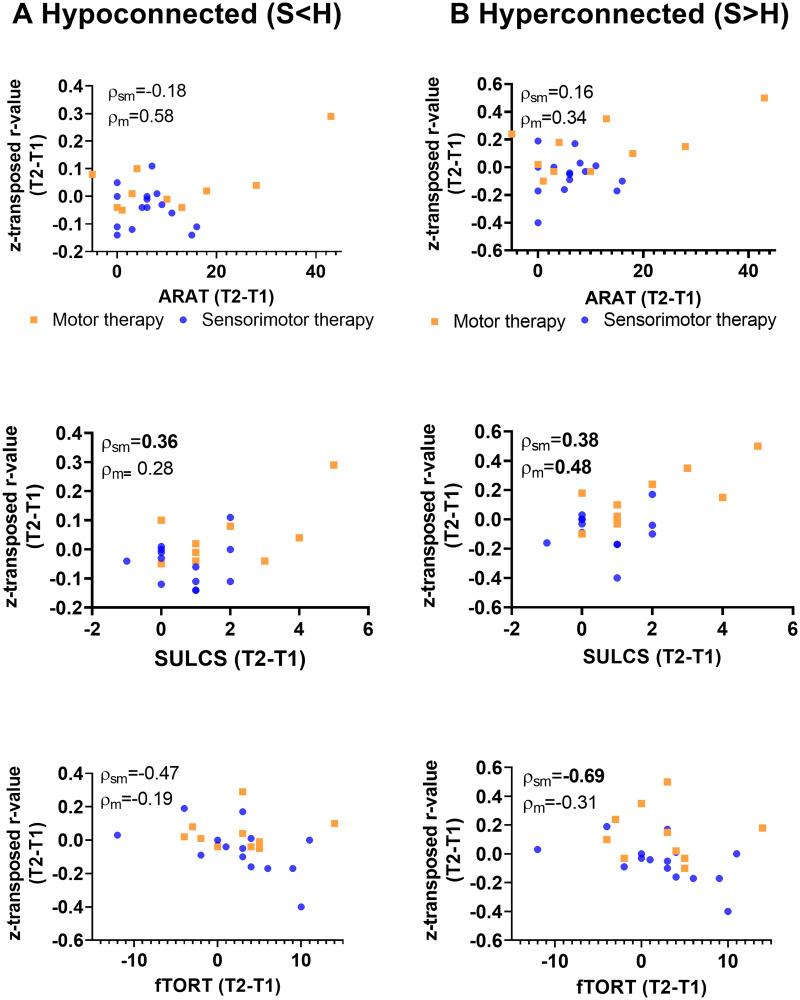
**Non-parametric partial correlations of pre−post-intervention improvements with alterations in contralesional intrahemipsheric functional connectivity indexes.** Scatterplots of raw change scores, rho-value indicates non-parametric (Spearman) partial correlation coefficient with correction for age and mean FD. Normalization of hypo-connectivity (due to pre-to-post increase in connectivity) in the contralesional hemisphere is associated with clinical improvements as assessed with SULCS for the sensorimotor group. Further, pre-to-post increases in contralesional functional connectivity among regions that already showed hyper-connectivity at baseline (stroke > healthy) were also associated with clinical improvements as assessed with SULCS for the motor therapy group, indicating that these patterns of hyper-connectivity were compensatory in nature. *P* < 0.1 indicated in bold; ARAT, action research arm test; SULCS, stroke upper limb capacity scale; fTORT, fucntional tactile objec recognition test.

Scatterplots of raw values with Spearman rho correlation coefficients corrected for age and mean FD are visualized in Fig. 5 for outcome measures showing significant and trend-level relations and are presented in full in Supplementary Table 5.

#### Secondary analysis

Similar results were found for secondary mixed model between-group analysis without mean FD or age as covariate. Associations between index and clinical change score show similar results, however less pronounced when not correcting for age and mean FD or correcting only for age.

No significant results were found for between-group nor brain**−**behaviour associations in unmasked ROIs (Supplementary Table 6).

## Discussion

In this exploratory pilot RCT, we identified differences in functional connectivity between healthy controls and stroke participants in the early rehabilitation phase post stroke. These differences included both connections showing hyper- as well as connections showing hypo-connectivity in stroke participants compared with healthy controls. Improvements of functional connectivity towards normalization were not significantly different between both therapy groups. However, trends towards a stronger increase in functional connectivity after motor therapy compared with sensorimotor therapy were identified. This may suggest that improvements in functional connectivity are associated with improvements in behavioural functional activity.

We have contrasted whole brain ROI-to-ROI functional connectivity of stroke patients with healthy controls and found significant differences in several connections with both patterns of hypo- and hyper-connectivity. Interestingly, regions showing functional hypo-connectivity in stroke patients were mostly located outside the typical sensorimotor network. In particular, most of these regions were located close to the secondary somatosensory cortex, which is in line with previous literature. Decreased functional connectivity in the temporal and occipital lobe was reported in stroke patients with somatosensory impairments compared with healthy controls^35^ Further, the left superior lingual gyrus was reported to show increases in brain activity between healthy controls and stroke patients during a shape discrimination task (task fmri).^36^ Co-activation of the inferior frontal gyrus during movement is related to disruption of the segregation of motor and attention related areas.^37^ On the other hand, connections showing functional hyper-connectivity were mainly located within the sensorimotor network. Notably, insular cortex showed both decreased and increased connections, indicating that insular connectivity may display an extensive pattern of reorganization after stroke. Previous research from our group already identified the insular cortex as a key region associated with somatosensory impairments post stroke using voxel-based lesion symptom mapping.^38^ Also a meta-analytic clustering approach^39^ identified the insular cortex as an important hub between sensorimotor areas and frontal and temporal areas.

Furthermore, we have contrasted changes in functional connectivity between both therapy groups and found that both groups might show a different therapy-induced effect. This was not in line with our hypothesis. For regions that showed hypo-connectivity in the stroke patients compared with controls, it might show that this pattern of hypo-connectivity was stabilized in the motor group after therapy. Importantly, these differences in alterations of functional connectivity were only found to be trending towards significance within the contralesional hemisphere. Within previous literature, the role of the contralesional hemisphere is inconclusive. On one hand, hyperactivity of the contralesional hemisphere was related to the absence of inhibitory activity of the lesioned hemisphere resulting in interhemispheric disbalance, which was associated with poor recovery.^40^ On the other hand, the contralesional hemisphere is described to have a supportive role into the recovery of function.^41^^,^^42^ Based on a longitudinal study, the role of the contralesional hemisphere is even described to be changing over time. A recovery-supportive role of the contralesional hemisphere is suggested at 2 weeks after stroke, changing to an inhibitory factor for recovery in the chronic phase post stroke.^43^ Hence, our findings from patients in the early rehabilitation phase may contribute to the supportive role of the contralateral hemisphere for motor recovery, and thus may provide new insight that this time window may be extended until the first months post stroke.

Group results suggest that the patients receiving sensorimotor therapy showed less pronounced normalisations of hypo-connectivity and (compensatory) increases in hyper-connectivity. However, also within this group, inter-individual differences in connectivity changes were associated with clinical improvements. Overall, changes in functional connectivity in the sensorimotor group may have been less pronounced due to some confounding factors induced by this therapy program. For example, the integration of somatosensory and motor function during sensorimotor therapy could induce cognitive sensorimotor interference in stroke patients. Competition between stimuli due to the engagement of the same neural circuits in both tasks could interfere with task performance and thus interfere with functional connectivity changes associated with task performance.^44^ Based on our between-group clinical findings, which suggests that the motor group might show significant better improvements in motor function compared with the sensorimotor group, we may suggest that the underlying brain recovery mechanism of the motor group is coherent with our clinical findings.

Finally, we investigated the brain**−**behaviour relationship between contralesional intrahemispheric functional connectivity changes and improvements in motor and somatosensory function. We found that the contralesional intrahemispheric connectivity index, which showed hyperactivity in stroke patients compared with healthy controls, may show the strongest associations with behavioural function, which can be expected since most of these regions were located within the sensorimotor network. Previous literature has shown that improvements in functional connectivity are restricted to the network of the specific function.^45^ Differences between both therapy groups could be identified with stronger associations with motor assessments for the motor therapy group and stronger associations with somatosensory assessments for the sensorimotor group. However, better improvements of clinical function were found for the motor group which could suggest the superiority of the underlying recovery mechanism for the motor group. Nevertheless, these results should be interpreted with caution due to small sample sizes of both therapy groups (*n* = 18 and 12).

An important element in rs-fMRI analysis in stroke patients is how to deal with the lesion. Previous studies often bypass this problem by investigating only brain regions, which are certainly unaffected, such as the contralesional hemisphere, or by only investigating patients with subcortical strokes.^16^^,^^42^^,^^46^ In this study, we included mainly patients with mixed cortical-subcortical strokes and took the lesion into account in two steps. First step was by applying enantiomorphic transformation during normalisation, which is a non-linear registration method that corrects the distorted signal of the brain lesion by using information from the contralesional undamaged homologous brain region.^47^ Secondly, we created two sets of ROIs, one set without taking into account the lesioned voxels (=unmasked) and one masked set, taking out all voxels of an ROI which were located into the lesioned area. Our findings were found in masked but not in the unmasked ROIs, which supports the role of lesioned voxels into the ROI. These lesioned voxels will impact more the averaged ROI value and thus reflect the overall impact of lesion on the functional impairment. Additionally, brain**−**behaviour associations were stronger in masked ROIs. This can implicate the role of the penumbra consisting of neighbouring voxels, which can take over the function of the lesioned voxels.^48^ Recovery of these non-lesioned neighbouring voxels could be the underlying mechanism of clinical recovery of upper limb function.^48^

Another important point to discuss it he choice to work with index values rather than specific connections. The rationale to create six index values (three for both hypo- and hyper-connectivity) is to reduce multiple comparison. We are aware of the limitations of this approach possibly overshadowing a strong effect of one specific connection. However, due to the small sample size, the large number of connections and the rather small changes in connectivity, we have decided not to perform an exploratory *post hoc* analysis investigating the relation between behavioural outcome changes and individual relevant connections. Performing this *post hoc* analysis would require a correction for multiple comparison, which will not reveal any significant result, even when taken very liberal, Furthermore, only connections that do show significant differences between healthy controls and stroke patients were selected for the inclusion into the index values and divided into hypo- or hyper-connectivity connections to overcome the problem of averaging out the effect. Since in literature a different role is described for both hemispheres independently (intrahemispheric) and interhemispheric, we also created these three subcategories.^6^^,^^40–43^ Additionally, differences were observed between connections that showed hyper- and hypo-connectivity in terms of location. The connections showing hyper-connectivity were mostly located into the sensorimotor network and those showing hypo-connectivity outside the sensorimotor network. In this way, we performed some kind of region-specific analysis.

We have to take into account some limitations of this study. First, we had a rather small sample size, however, this was in line with previous phase II proof of mechanism studies in literature. Therefore, subgroup association analysis should be interpreted with caution (*n* = 9 and 15). To further deal with the power issue, we created index values limiting multiple testing. Second, we have measured patients at admission to rehabilitation centre, which can induce selection bias and we do not have a fixed time point at which every patient was assessed. However, we limited the selection bias by only recruiting patients within the first 8 weeks post stroke in order to include all patients within the same early rehabilitation time window. Third, differences in baseline mean FD were found between healthy controls and both stroke therapy groups which could have influenced the results. However, we have corrected for mean FD in all analysis to minimize the effect of these differences. Forth, differences were found at baseline for age and lesion side between both therapy groups. The sensorimotor group was older and showed more right hemispheric lesions. Since age has been shown to be an influencing factor for recovery after stroke and the impact of lesion side is still inconclusive, we have corrected for age but not for lesion side.^49^ Finally, despite the evidence for the role of the cerebellum into sensorimotor function, we were not able to include cerebellar regions in our analyses due to technical limitations of the resting-state scanning protocol (i.e. cerebellum was only partially included in the field of view). Further research should include both cortical, subcortical as well as cerebellar regions into their analysis.

To conclude, we found that stroke patients showed both hyper- and hypo-connected functional connectivity within the contralesional intrahemispheric network compared with healthy controls. Stronger normalization of hypo-connectivity and a stronger increase in compensatory hyper-connectivity of the contralesional hemisphere were found after motor therapy compared with sensorimotor therapy. This underlying recovery mechanism could be suggested as the upper limb motor recovery strategy for patients within the first months’ post stroke.

## Supplementary material

Supplementary material is available at *Brain Communications* online.

## Supplementary Material

fcab074_Supplementary_Data

## Notes

This trial is registered at clinicaltrails.gov NCT03236376.

## Abbreviations

ARAT =: action research arm test
AUC =: area under the curve
BOLD =: blood oxygen level dependent
Em-NSA =: Erasmus modified Nottingham sensory assessment
FD =: frame-wise displacement
FDR-correction =: false discovery rate correction
FLAIR =: fluid-attenuated inversion recovery
FMA-UE =: Fugl-Meyer Assessment- upper extremity
FOV =: field of view
fTORT =: functional tactile object recognition test
MNI =: Montreal neurological institute
MOCA =: Montreal cognitive assessment
PTT =: perceptual threshold of touch
ROI =: region of interest
rs-fMRI =: resting-state functional MRI
S<H =: connections showing hypo-connectivity in stroke patients compared with healthy controls
S>H =: connections showing hyper-connectivity in stroke patients compared with healthy controls
S2 =: secondary somatosensory cortex
SE =: standardized error
SULCS =: Stroke upper limb capacity scale
T1 =: baseline assessment
T2 =: post-intervention assessment
T3 =: follow-up assessment
TDT =: texture discrimination test
TE =: Echo time
TR =: repetition time
WPST =: wrist position sense test
