# Supplementary material for: Brain connectivity alterations after additional sensorimotor or motor therapy for the upper limb in the early-phase post stroke: a randomized controlled trial
Source: Brain Commun. 2021 Apr 12;3(2):fcab074. doi: 10.1093/braincomms/fcab074 (PMC8072522; doi:10.1093/braincomms/fcab074)
Supplement: fcab074_Supplementary_Data [file fcab074_supplementary_data.pdf]

SUPPLEMENTARY FILES

Supplementary fig 1. Motion parameters. Percentage of scrubbed volumes and mean frame wise displacement (mean FD) for alle stroke particapants at baseline (T1), post-intervention (T2) and follow-up (T3) and healthy controls at baseline. Significant difference was found between sensorimotor group and both motor group and healthy controls

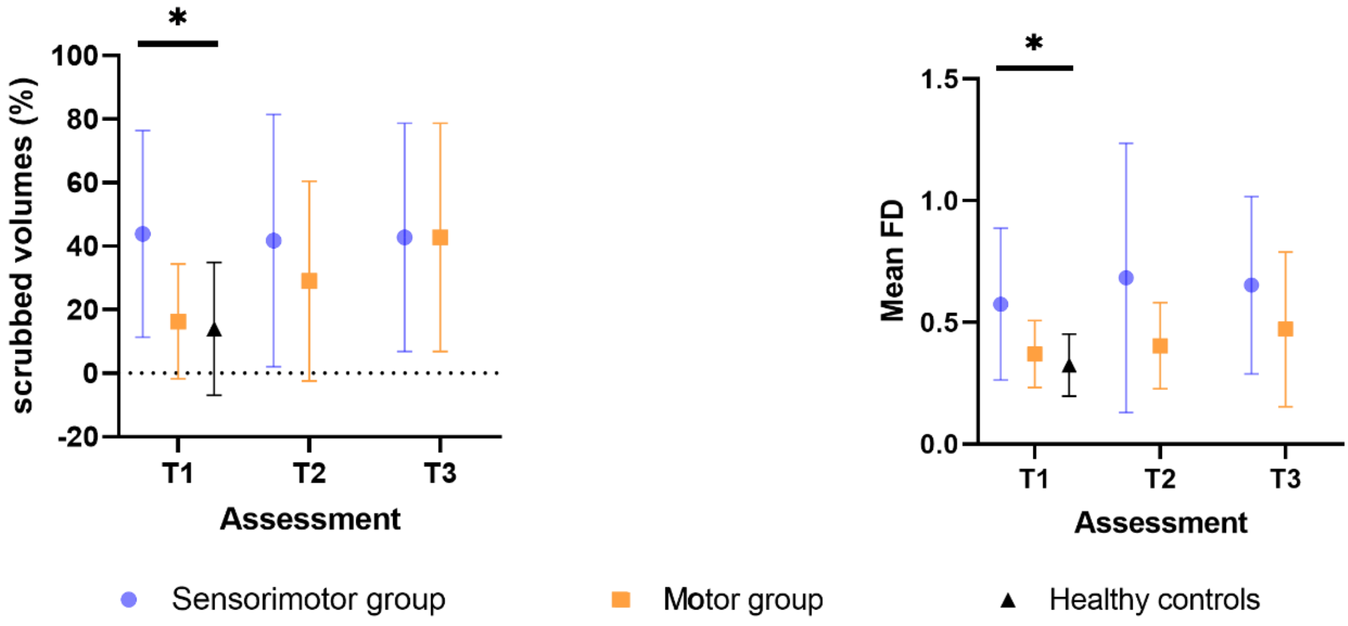

.

Supplementary Fig 2. Visualisation of regions that show connections with altered functional connectivity between healthy controls and stroke subjects. Abbreviations of region labels can be found in supplementary table 1.

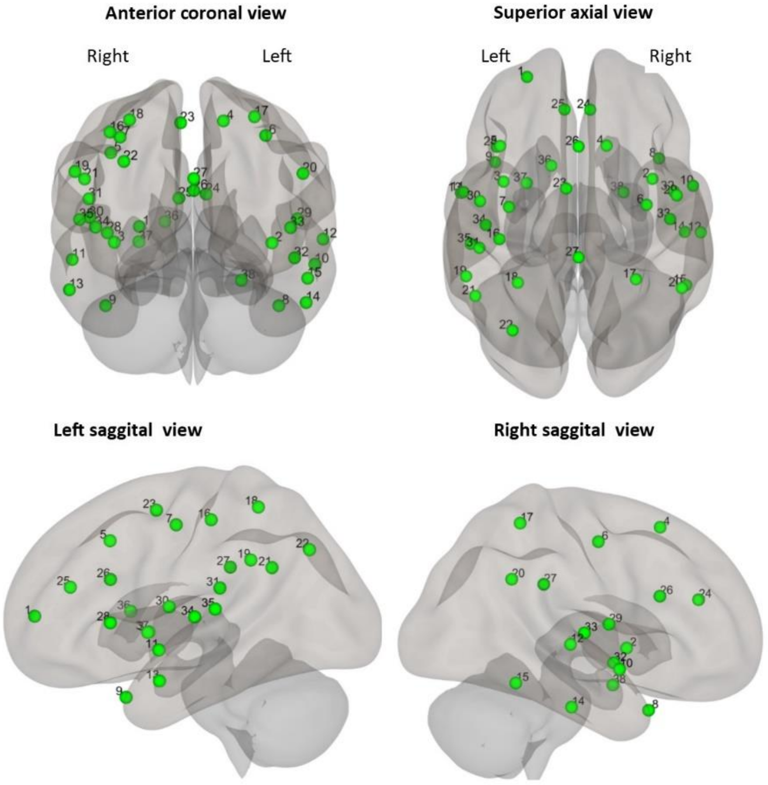

ROI Labels

Left		Right	
1	FP l		
3	IC l	2	IC r
5	MidFG l	4	SFG r
7	PreCG l	6	PreCG r
9	TP l	8	TP r
11	aSTG l	10	aSTG r
13	aMTG l	12	pSTG r
16	PostCG l	14	pITG r
18	SPL l	15	toITG r
19	pSMG l	17	SPL r
21	AG l	20	AG r
22	sLOC l	26	AC
23	SMA L	27	PC
25	PaCiG l	24	PaCiG r
28	FO l		
30	CO l	29	CO r
31	PO l	32	PP r
34	HG l	33	HG r
35	PT l	38	Amygdala r
36	Caudate l		
37	Putamen l		

Supplementary table 1. Regions of Interest H vs S

FP l	Frontal Pole Left
IC r	Insular Cortex Right
IC l	Insular Cortex Left
SFG r	Superior Frontal Gyrus Right
MidFG l	Middle Frontal Gyrus Left
PreCG r	Precentral Gyrus Right
PreCG l	Precentral Gyrus Left
TP r	Temporal Pole Right
TP l	Temporal Pole Left
aSTG r	Superior Temporal Gyrus, anterior division Right
aSTG l	Superior Temporal Gyrus, anterior division Left
pSTG r	Superior Temporal Gyrus, posterior division Right
aMTG l	Middle Temporal Gyrus, anterior division Left
pITG r	Inferior Temporal Gyrus, posterior division Right
toITG r	Inferior Temporal Gyrus, temporo-occipital part Right
PostCG l	Postcentral Gyrus Left
SPL r	Superior Parietal Lobule Right
SPL l	Superior Parietal Lobule Left
pSMG l	Supramarginal Gyrus, posterior division Left
AG r	Angular Gyrus Right
AG l	Angular Gyrus Left
sLOC l	Lateral Occipital Cortex, superior division Left
SMA L	Juxtapositional Lobule Cortex -formerly Supplementary Motor Cortex- Left
PaCiG r	Paracingulate Gyrus Right
PaCiG l	Paracingulate Gyrus Left
AC	Cingulate Gyrus, anterior division
PC	Cingulate Gyrus, posterior division
FO l	Frontal Operculum Cortex Left
CO r	Central Opercular Cortex Right
CO l	Central Opercular Cortex Left
PO l	Parietal Operculum Cortex Left
PP r	Planum Polare Right
HG r	Heschl"s Gyrus Right
HG l	Heschl"s Gyrus Left
PT l	Planum Temporale Left
Caudate l	Caudate Left
Putamen l	Putamen Left
Amygdala r	Amygdala Right

Supplementary table 2. Between group comparison of an intervention effect corrected for age

Motor function

		**ARAT** /57	**FMA** /66	**SULCS** /10	**ABILDHAND** (logits)
**T2-T1**	Sensorimotor group	4.87 (2.32)	5.99 (2.06)	0.84 (0.38)	4.86(1.42)
	Motor group	11.06 (2.46)	14.65 (2.19)	1.87 (0.37)	4.13 (1.47)
	p-value	**0.08**	**0.01***	**0.08**	0.74
	95%CI	(-13.24-0.87)	(-15.03--2.29)	(-2.16-0.11)	(-3.79 -5.24)
**T3-T2**	Sensorimotor group	2.00(1.42)	1.20 (1.65)	0.28 (0.37)	5.00(1.55)
	Motor group	6.07 (1.53)	2.98 (1.72)	0.75 (0.39)	0.28 (1.71)
	p-value	**0.08**	0.48	0.41	**0.06**
	95%CI	(-8.67-0.46)	(-6.92-3.35)	(-1.61-0.68)	(-0.28-9.68)
**T3-T1**	Sensorimotor group	7.65 (2.73)	6.75 (2.29)	0.90 (0.45)	9.12 (1.67)
	Motor group	16.32 (2.98)	17.38 (2.37)	2.60 (0.45)	4.26 (1.76)
	p-value	**0.04**	**0.003***	**0.02**	**0.07**
	95%CI	(-17.00--0.34)	(-17.50--3.76)	(-3.04--0.36)	(-0.32-10.05)

Somatosensory function

		**Em-NSA** /40	**PTT** /10mA	**TDT**-AUC	**WPST** total error degrees	**WPST** mean error degrees	**fTORT /42**
**T2-T1**	Sensorimotor group	1.48(1.37)	-1.15 (0.54)	10.18 (6.13)	-56.43 (28.50)	-1.83 (1.32)	2.41 (1.36)
	Motor group	2.01 (1.36)	-0.15 (0.57)	5.11 (6.41)	-62.08 (30.88)	-3.51 (1.41)	3.39 (1.43)
	p-value	0.79	0.22	0.58	0.90	0.40	0.63
	95%CI	(-4.65-3.57)	(-2.62 -0.62)	(-13.53-23.65)	(-83-94)	(-2.35-5.70)	(-6.02-2.65)
		0.85 (1.09)	-0.41 (0.39)	-0.22 (4.53)	-7.61 (22.22)	-0.07 (1.08)	0.08 (0.99)

**T3-T2**	Sensorimotor group						
	Motor group	1.31 (1.22)	-0.37 (0.44)	-2.14 (4.89)	-13.54 (24.00)	-0.90 (1.15)	0.40 (1.06)
	p-value	0.79	0.94	0.79	0.87	0.62	0.83
	95%CI	(-3.94-3.03)	(-1.30 -1.20)	(-12.46-16.29)	(-65-77)	(-2.50 -4.16)	(-3.41 -2.75)
**T3-T1**	Sensorimotor group	1.57 (1.47)	-1.39 (0.44)	10.05 (7.00)	-33.13 (32.30)	-1.83 (1.32)	2.41 (1.43)
	Motor group	3.66 (1.49)	-1.14 (0.50)	2.45 (7.52)	-94.46 (34.65)	-3.51 (1.41)	4.09 (1.53)
	p-value	0.34	0.73	0.48	0.22	0.40	0.44
	95%CI	(-6.49-2.32)	(-1.63-1.15)	(-13.75-8.94)	(-37 -160)	(-2.35-5.70)	(-6.02-2.65)

Estimated mean and standard error of changes scores (T2-T1, T3-T2, T3-T1) are presented for both groups; p-values based on mixed models with age stroke onset (years) as covariate to evaluate differences between the change scores of both groups. Correction for multiple comparison was set on p<0.02. ARAT: action research arm test, FMA-UE: Fugl-Meyer assessment upper extremity section, SULCS: stroke upper limb capacity scale, Em-NSA: Erasmus modification of Nottingham sensory assessment, PTT: perceptual threshold of touch, TDT: texture discrimination test, AUC: area under curve, WPST: wrist position sense test, fTORT: functional tactile object recognition test. Adopted from [17]

Supplementary table 3a. ROI-to-ROI connections showing hypoconnectivity

Seed ROI		Connected ROI		T(44)	p-FDR corrected
AC		TP	l	-3.86	0.0081
AC		Amygdala	r	-4.09	0.0067
AC		TP	r	-5.05	0.0008
AG	l	pITG	r	-4.29	0.01
aSTG	r	CO	r	-3.7	0.009
aSTG	r	IC	r	-3.81	0.0074
aSTG	r	HG	r	-3.81	0.0074
aSTG	r	IC	l	-4	0.0074
aSTG	r	PP	r	-4.09	0.0074
aSTG	l	aSTG	r	-3.88	0.0051
aSTG	l	IC	l	-3.89	0.0051
aSTG	l	HG	l	-3.95	0.0051
aSTG	l	CO	r	-4.31	0.0024
aSTG	l	IC	r	-4.77	0.0007
aSTG	l	HG	r	-4.83	0.0007
aSTG	l	PP	r	-6.24	0
HG	l	PP	r	-4.44	0.0031
HG	l	aSTG	r	-4.46	0.0031
IC	r	PT	l	-4.48	0.0028
pITG	r	PC		-4.65	0.0016
PP	r	PT	l	-4.57	0.0021
pSMG	l	pITG	r	-5.27	0.0004
PT	l	aMTG	l	-3.98	0.0088
TP	r	PaCiG	r	-4.72	0.0013

Regions of Interest (ROI) analysis comparing Healthy with stroke participants; mean framewise displacement as nuissance regressor. Overview of ROI labels can be found in supplementary table 1.

Supplementary table 3b. ROI-to-ROI connections showing hyperconnectivity

Seed ROI		Connected ROI		T(44)	p-FDR corrected
AC		AG	r	4.07	0.0067
AC		Caudate	l	3.84	0.0081
Caudate	l	PaCiG	r	3.81	0.0089
Caudate	l	Putamen	l	3.88	0.0089
Caudate	l	PaCiG	l	4.03	0.0089
Caudate	l	SFG	r	4.33	0.0089
CO	l	PO	l	4.24	0.0059
CO	l	FO	l	5.1	0.0007
FO	l	HG	l	4.09	0.0062
FO	l	IC	l	4.7	0.0014
MidFG	l	FP	l	4.14	0.0079
MidFG	l	sLOC	l	4.51	0.0049
PostCG	l	PreCG	l	4.31	0.0095
PreCG	r	SPL	r	4.34	0.0086
PreCG	l	SPL	l	4.52	0.0047
pSTG	r	toITG	r	4.37	0.0077
SPL	l	SMA	L	4.21	0.0064

Regions of Interest (ROI) analysis comparing Healthy with stroke participants; mean framewise displacement as nuisance regressor. Overview of ROI labels can be found in supplementary table 1.

Supplementary table 4. Between group comparison of an intervention effect

Masked ROIs

		T2-T1 Sensorimotor group	Motor group	p-value	T (df)	T3-T1 Sensorimotor group	Motor group	p-value	T(df)
**Strokes < Healthy**	interhemispheric connectivity index	-0.004 (0.041)	0.100 (0.050)	0.13	T(28)=-1.54	0.028 (0.036)	0.098 (0.042)	0.23	T(25)=-1.23
	ispilesional intrahemispheric index	-0.040 (0.037)	0.039 (0.045)	0.2	T(27) =-1.32	-0.036(0.042)	0.028 (0.048)	0.35	T(25 =-0.96
	contralesional intrahemispheric index	-0.084 (0.037)	0.071 (0.045)	**0.02**	T(25)=-2.56	-0.090 (0.043)	0.111(0.50)	**0.01**	T(24)=-2.96
**Strokes > Healthy**	interhemispheric connectivity index	0.010 (0.069)	0.138 (0.085)	0.27	T(28) =-1.12	0.051 (0.074)	0.079 (0.085)	0.81	T(25) =-0.24
	ispilesional intrahemispheric index	-0.048 (0.047)	-0.014 (0.057)	0.66	T(27)= -0.44	-0.069 (0.055)	-0.001 (0.064)	0.43	T(25) = -0.81
	contralesional intrahemispheric index	-0.030 (0.043)	0.113 (0.053)	**0.06**	T(27)= -2.00	0.035 (0.059)	0.156 (0.069)	0.21	T(24) = -1.30

Mixed models with change scores of masked connectivity index with age and mean FD as nuisance covariates, estimated marginal means with standard error are reported for each group and each index value. P-value for multiple testing is set on $p < 0.003$. mean FD scores of both time points was averaged to include into mixed model analysis; T1: baseline; T2: post-intervention; T3: follow-up; ROI: region of interest T: t-statistic; df: degrees of freedom

Supplementary table 5. subgroup analysis: non parametric partial correlations of pre-post intervention improvements

MOTOR

	ARAT		FMA-UE		SULCS	
	**SM**	**M**	**SM**	**M**	**SM**	**M**
**Contralesional intrahemispheric (T2-T1)**						
**S<H**	0.45	0.37	-0.08	**0.75**	-0.18	0.50
**S>H**	0.16	0.34	0.21	0.62	**0.38**	**0.48**

SOMATOSENSORY

	Em-NSA		PTT		TDT		WPST		fTORT	
	**SM**	**M**	**SM**	**M**	**SM**	**M**	**SM**	**M**	**SM**	**M**
**Contralesional intrahemispheric (T2-T1)**										
**S<H**	0.19	0.07	-0.29	0.12	0.22	0.04	**0.57**	**-0.67**	0.06	0.41
**S>H**	**0.76**	0.02	-0.35	-0.28	**0.54**	0.45	0.06	-0.03	**-0.69**	-0.31

Subgroup non-parametric (spearman) partial correlation with correction for age and mean framewise displacement (FD). Mean FD of T1 and T2 was averaged when included as nuisance regressor into the difference in difference analysis. $p<0.1$ indicated in bold; SM: sensorimotor group; M: motor group; ARAT: action research arm test; FMA-UE: Fugl-Meyer assessment upper extremity part; SULCS: stroke upper limb capacity scale; Em-NSA: Erasmus modification of Nottingham sensory assessment; PTT: perceptual threshold of touch; TDT: texture discrimination test; WPST: wrist position sense test; fTORT: functional tactile object recognition test

Supplementary table 6. Between group comparison of an intervention effect

Unmasked ROIs

		T2-T1 Sensorimotor group	Motor group	p-value	T (df)	T3-T1 Sensorimotor group	Motor group	p-value	T(df)
**Strokes < Healthy**	interhemispheric connectivity index	-0.001(0.024)	0.036 (0.030)	0.37	T(28)=-0.91	-0.003 (0.028)	0.039 (0.033)	0.35	T(24)=-0.95
	ispilesional intrahemispheric index	0.000(0.027)	0.026 (0.034)	0.57	T(27)=-0.58	0.000(0.026)	0.021(0.030)	0.61	T(25)=-0.52
	contralesional intrahemispheric index	-0.042 (0.028)	0.016 (0.034)	0.21	T(28)=-1.27	-0.027 (0.030)	0.033 (0.035)	0.22	T(25) =-1.26
**Strokes > Healthy**	interhemispheric connectivity index	0.100 (0.090)	-0.061 (0.110)	0.28	T(24) =1.10	-0.047 (0.079)	-0.001 (0.092)	0.72	T(25) =-0.36
	ispilesional intrahemispheric index	0.020 (0.056)	0.051 (0.069)	0.74	T(25)=-0.33	-0.001 (0.043)	-0.009 (0.050)	0.91	T(23)=0.11
	contralesional intrahemispheric index	-0.044(0.046)	-0.056(0.056)	0.87	T(27)=0.16	.000(0.050)	-0.006(0.058)	0.94	T(24)=0.08

Mixed models with change scores of unmasked connectivity index with age and mean FD as nuisance covariates, estimated marginal means with standard error are reported for each group and each index value. P-value for multiple testing is set on p < 0.003. mean FD scores of both time points was averaged to include into mixed model analysis; T1: baseline; T2: post-intervention; T3: follow-up; ROI: region of interest T: t-statistic; df: degrees of freedom
